# Loss of tumor suppressor mir-203 mediates overexpression of LIM and SH3 Protein 1 (LASP1) in high-risk prostate cancer thereby increasing cell proliferation and migration

**DOI:** 10.18632/oncotarget.1928

**Published:** 2014-04-27

**Authors:** Amelie Hailer, Thomas GP Grunewald, Martin Orth, Cora Reiss, Burkhard Kneitz, Martin Spahn, Elke Butt

**Affiliations:** ^1^ Institute for Clinical Biochemistry and Pathobiochemistry, University Clinic of Wuerzburg, Grombuehlstrasse 12, 97080 Wuerzburg, Germany; ^2^ INSERM Unit 830, Genetics and Biology of Cancers, Institute Curie Research Center, 26 rue d'Ulm, 75248 Paris, France; ^3^ Urology and Pediatric Urology, University Clinic of Wuerzburg, Oberduerrbacher Strasse 6, 97080 Wuerzburg, Germany

**Keywords:** LASP1, prostate cancer, mir-203, PSA, LNCaP

## Abstract

Several studies have linked overexpression of the LIM and SH3 domain protein 1 (LASP1) to progression of breast, colon, liver, and bladder cancer. However, its expression pattern and role in human prostate cancer (PCa) remained largely undefined.

Analysis of published microarray data revealed a significant overexpression of LASP1 in PCa metastases compared to parental primary tumors and normal prostate epithelial cells. Subsequent gene-set enrichment analysis comparing LASP1-high and -low PCa identified an association of LASP1 with genes involved in locomotory behavior and chemokine signaling. These bioinformatic predictions were confirmed *in vitro* as the inducible short hairpin RNA-mediated LASP1 knockdown impaired migration and proliferation in LNCaP prostate cancer cells.

By immunohistochemical staining and semi-quantitative image analysis of whole tissue sections we found an enhanced expression of LASP1 in primary PCa and lymph node metastases over benign prostatic hyperplasia. Strong cytosolic and nuclear LASP1 immunoreactivity correlated with PSA progression. Conversely, qRT-PCR analyses for mir-203, which is a known translational suppressor of LASP1 in matched RNA samples revealed an inverse correlation of LASP1 protein and mir-203 expression. Collectively, our results suggest that loss of mir-203 expression and thus uncontrolled LASP1 overexpression might drive progression of PCa.

## INTRODUCTION

Prostate cancer (PCa) is the most frequent cancer of men in the western world [1]. Although many PCa are rather indolent tumors and remain clinically stable for many years or even decades and do not require any treatment, more aggressive PCa subtypes metastasize early and are associated with dismal outcome [2-4]. In 1986, introduction of prostate specific antigen (PSA) testing has significantly improved early diagnosis of PCa [5]. However, although high serum PSA levels may correlate with PCa aggressiveness [6], PSA testing has caused a stage shift to less aggressive PCa. Over-detection and over-treatment are the main drawbacks of PSA-testing and unintentionally affect patients' quality of life [7]. Consequently, there is an urgent need for prognostic biomarkers to discriminate indolent from highly aggressive PCa in order to better guide an individual patient's treatment.

Recently, Erho et al. [8] developed and validated a PCa genomic classifier set with 22 markers that predicts metastatic progression better than clinicopathologic variables. The LIM and SH3 protein 1 (LASP1) is one of these markers.

LASP1 is a nucleo-cytosolic shuttling protein involved in migration, adhesion, proliferation and cell cycle progression of many cancers [9]. LASP1 was initially identified from a cDNA library of breast cancer metastases and the corresponding protein is overexpressed in more than 50% of all breast cancers [10-12]. Besides its function as a structural scaffolding protein at sites of actin assembly such as invadopodia and membrane ruffles [13], LASP1 likely acts as a signaling molecule transducing information from the cytoplasm into the nucleus [14]. LASP1 is expressed in virtually all normal tissues [9], but overexpressed in many cancer entities such as the aggressive pediatric brain tumor medulloblastoma [15] as well as breast [12], ovarian [16] and colorectal carcinoma [17]. Moreover, LASP1 overexpression correlates with adverse outcome in these cancer entities suggesting an oncogenic function of LASP1 [12, 15, 17]. Expression of LASP1 is regulated i) by tumor suppressor p53 on the genomic level as shown for hepatocellular carcinoma [18] and ii) on the protein level by microRNA mir-203 as described for esophageal squamous cell carcinoma [19], breast cancer [20], and PCa [21, 22].

Here, we investigated the LASP1 expression pattern in a large series of surgically treated high-risk PCa samples (n=161) and correlated LASP1 protein levels with mir-203 expression levels in a subset of the same tumors (n=138). In addition, we investigated the effect of RNA interference mediated LASP1 knockdown in a metastatic PCa cell line.

Our data demonstrate for the first time that LASP1 is overexpressed in a subset of high-risk PCa and that this expression correlates with PSA progression. qRT-PCR revealed a correlation between high LASP1 protein levels and reduced mir-203 expression in the PCa tissue samples. Cell culture experiments underline the more proliferative and migratory PCa phenotype in high LASP1 expressing cells.

These data might represent a first step toward characterizing LASP1 as a promising novel candidate biomarker to discriminate indolent from aggressive PCa.

## RESULTS

### LASP1 mRNA expression is increased in prostate cancer metastases and is associated with pathways involved in cell migration

**Figure 1:**
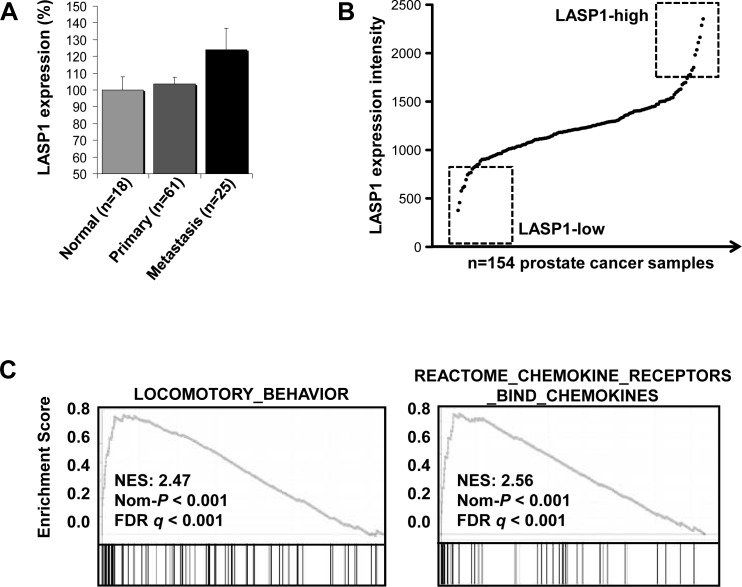
LASP1 mRNA expression is increased in prostate cancer metastases and is associated with pathways involved in cell migration A: LASP1 is significantly overexpressed in PCa metastases compared to parental primary tumors and normal prostate epithelial cells (p=0.028; Student's t test metastasis normal and primary) B: Gene expression signatures in the top 10 LASP1-high versus top 10 LASP1-low PCa samples by gene-set enrichment analysis (GSEA). C: GSEA analysis of the PCa microarray data, showing significant enrichment of genes involved in locomotory behaviour and chemokine signaling.

As LASP1 is overexpressed in several cancer entities [15-17], we assessed the LASP1 mRNA expression pattern in publicly available microarray datasets. Specifically, microarray data of primary PCa (n=61) and PCa metastases (n=25) were compared with normal prostate tissue (n=18). As displayed in Figure 1A, LASP1 is significantly (p=0.028) overexpressed in PCa metastases compared to normal tissues and the primary tumor.

**Table 1:** Patient characteristics (n=161)

Parameters	Median
Age at surgery, years (range)	65.9 (43-81)
Mean follow-up, months (range)	47.14 (1-105)
Nuclear LASP1 positivity	56 (34.78%)
LASP1-IRS positivity	39 (24.22%)
Clinical failure/clinical recurrence	19 (11.80%)
Mean preoperative PSA (ng/ml) (range)	48.07 (20-160)
Death for any reason	22 (13.66%)
Cancer related death (CRD)	10 (6.21%)
Cancer specific survival, months (range)	43.7 (14-63)
Biochemical progression/PSA progression	41 (25.47%)
Average time to PSA progression, months (range)	22.8 (1-54)
Average time to clinical progression, months (range)	26.79 (3-89)
Gleason score	
6	3 (1.86%)
7	49 (30.43%)
8	52 (32.3%)
9	43 (26.71%)
10	14 (8.7%)
Pathological tumor stage	
pT2	23 (14.29%)
pT3a	44 (27.33%)
pT3b	67 (41.61%)
pT4	27 (16.77%)
Lymph node positive	59 (36.65%)

To investigate LASP1 correlated pathways, we analyzed a large PCa microarray study (n=154) for LASP1 expression. As displayed in Figure 1B, LASP1 is moderately expressed in most PCa samples but appears to be overexpressed in about 15% of PCa samples (designated as LASP1-high). Subsequently, we compared the gene expression signatures in the top 10 LASP1-high versus top 10 LASP1-low PCa samples (Figure 1B and Supplemental Table 1) by gene-set enrichment analysis (GSEA). GSEA revealed that LASP1 overexpression in clinical PCa samples is strongly correlated (p<0.001) with gene signatures involved in locomotory behaviour and chemokine signaling (Figure 1C). Collectively, these data indicate that LASP1 overexpression is associated with a more aggressive PCa phenotype.

### LASP1 protein expression is elevated in metastatic prostate cancer

**Figure 2:**
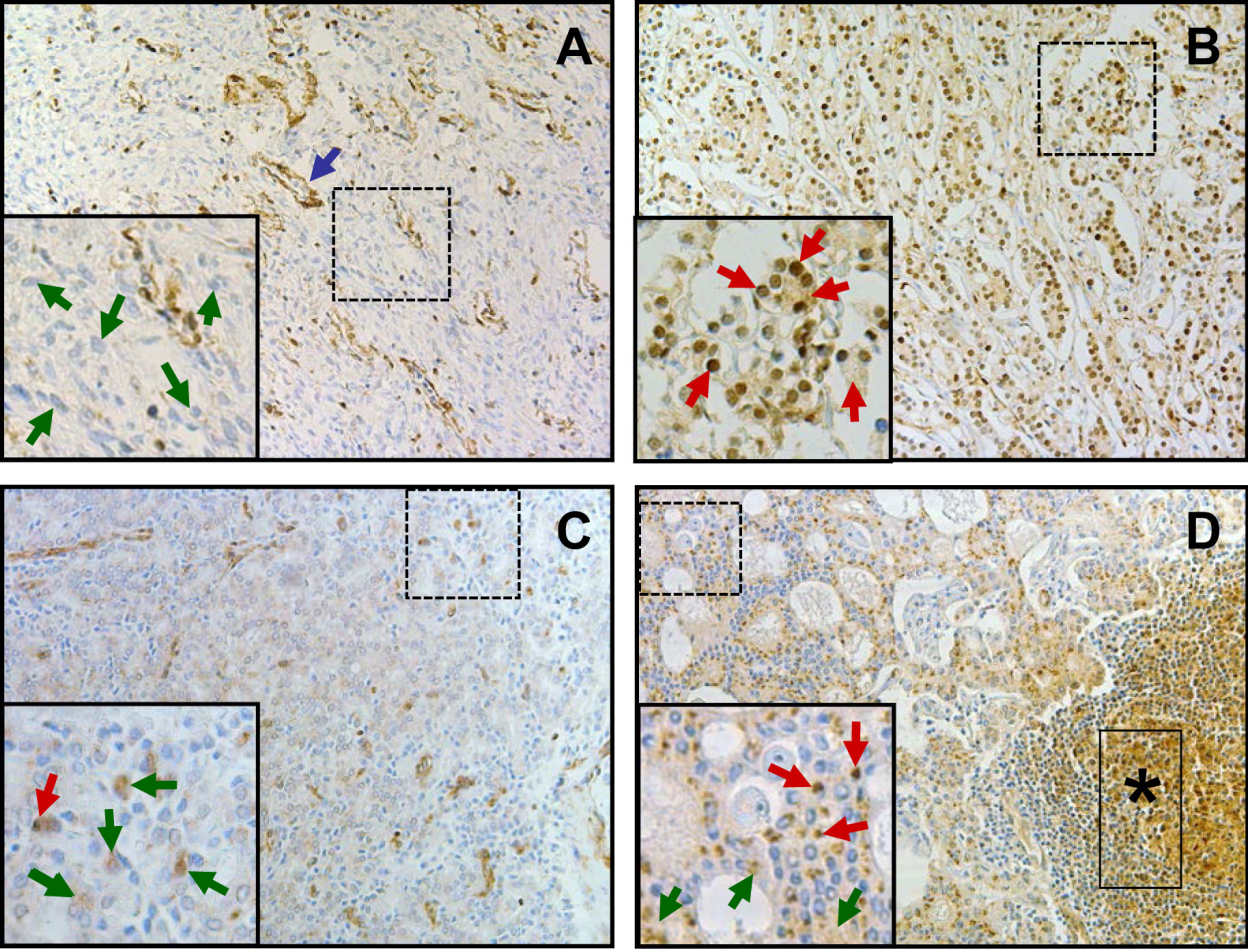
LASP1 protein expression is elevated in metastatic prostate cancer A: Representative immunostaining of BPH: most hyperplastic cells are LASP1 negative, both, for cytosol and nucleus (green arrows). Smooth muscle cells of blood vessels are positive for LASP1 (blue arrow). B: Representative PCa immunostaining (Gleason 8): most cells display positive LASP1 staining, both, for cytosol (IRS 4) and nuclei (red arrows). C and D: Prostate cancer (Gleason 8) with corresponding LNM, respectively: in PCa most cells are weak positive for cytosolic LASP1 staining (IRS 1) (green arrows). Few cells show additional positive nuclear LASP1 staining (red arrow). Compared to the primary tumor shown in C, in LNM (D) the overall cytosolic LASP1 staining (IRS 4) (green arrows) and the amount of cells with additional LASP1 positive nuclei (red arrows) are increased. LASP1 positive lymphocytes are marked with a black asterisk. (DAB, brown, magnification x40)

To validate our findings on LASP1 overexpression in a subset of PCa samples on protein level, we investigated the LASP1 protein expression pattern by immuno-histochemistry (IHC) in specimens from 15 benign prostatic hyperplasias (BPH), 161 high-risk PCa derived from patients with pre-treatment PSA >20 ng/ml who underwent radical prostatectomy, and 17 corresponding lymph node metastases (LNM). Representative samples for the observed LASP1 immunoreactivity are shown in Figure 2. Analysis of the Immune Reactive Scores (IRS) revealed that the median expression of LASP1 increases from BPH (IRS 2.0 ± 1) to PCa (IRS 3.0 ± 2) and LNM (IRS 4.0 ± 1). Interestingly, only PCa and LNM showed very high IRS values up to 12 while in BPH, the IRS maximum is 6. Accordingly, a low cytosolic LASP1 expression correlated with negative/low nuclear staining and a high cytosolic expression with high nuclear LASP1 immunoreactivity (p=0.0001, Table 2). These analyses confirmed an increase of LASP1 protein levels in metastatic high-risk PCa.

**Table 2:** LASP1 and mir-203 expression in BPH, PCa and LNM

	Positive LASP1 nucleus (≥10%)	Mean Nuclear staining (range)	Positive LASP1 (IRS>5)	Median IRS (range)	ΔCT mir-203 (subset)
BPH (n=15)	1 (6.7%)	3.3 (0-13)	1 (6.7%)	2 (0-6)	−0.54 ± 0.16 (15)
PCa (n=161)	56 (34.8%)	16.3 (0-95)	39 (24.2%)	3 (0-12)	−1.54 ± 0.12 (138)
LNM (n=17)	5 (29.4%)	12.5 (2-50)	2 (11.8%)	4 (1-11)	−2.39 ± 0.26 (12)

### Silencing of LASP1 impairs proliferation and migration of prostate cancer cells in vitro

**Figure 3:**
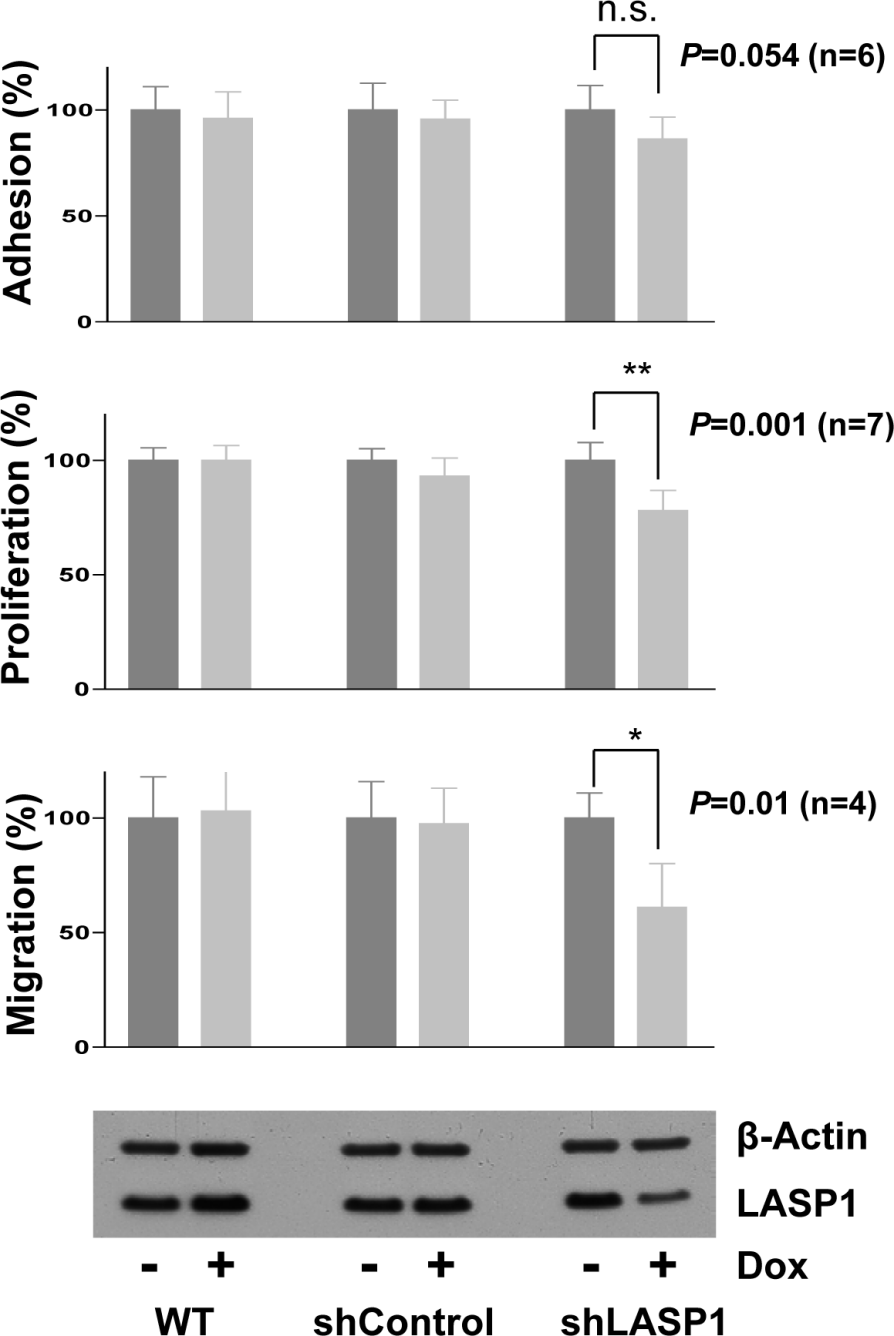
Silencing of LASP1 impairs proliferation and migration of prostate cancer cells *in vitro* Used were non-transfected LNCaP wild type cells (WT) and cells stably transfected with control shRNA or shRNA against LASP1. Adhesion: After 4 days LASP1 knockdown, cells were seeded in 48-well plates and incubated for 7.5h. Adherent cells were counted using CellTiterGlo^®^. Proliferation: Cells were seeded in T25 flasks. Knockdown was induced with doxycycline (Dox) and cells were counted after 4 days. Migration: After 4 days LASP1 knockdown, cells were seeded in modified Boyden chambers and incubated for 4 h. Migrated cells were fixed, stained with crystal violet and absorbance was measured. Bar plots represent mean ±SEM; p, Student's *t* test, *versus* control. LASP1 knockdown efficiency was controlled by Western blot. Actin is shown as loading control.

To functionally assess the role of LASP1 in high-risk PCa, we used the LNCaP cell line, which is commonly used as an *in vitro* model for metastasized PCa [23]. LNCaP cells were stably transfected with inducible shRNA against LASP1 or a control shRNA. Doxycyclin-induced LASP1 knockdown was confirmed for every experiment by Western blot (WB) and showed an average silencing of LASP1 of about 50% (Figure 3, lowest panel).

Proliferation was assessed by cell counting and revealed a significant inhibition of cell proliferation up to 31% upon LASP1 silencing (Figure 3). Similar results were obtained using a CellTiter-Glo® Luminescent assay (data not shown).

Since LASP1 has been shown to promote cell motility and metastasis in other tumor entities [17, 24] we analyzed cell migration and adhesion of LNCaP cells before and after LASP1 silencing with a modified Boyden-chamber and an adhesion assay, respectively. We observed a strong reduction in migratory potential by 39% upon LASP1 silencing but no significant effect on adhesion (Figure 3). Taken together, these data provide evidence that LASP1 is functionally involved in PCa cell proliferation and migration.

### Cytosolic and nuclear LASP positivity correlate with PSA progress

**Figure 4:**
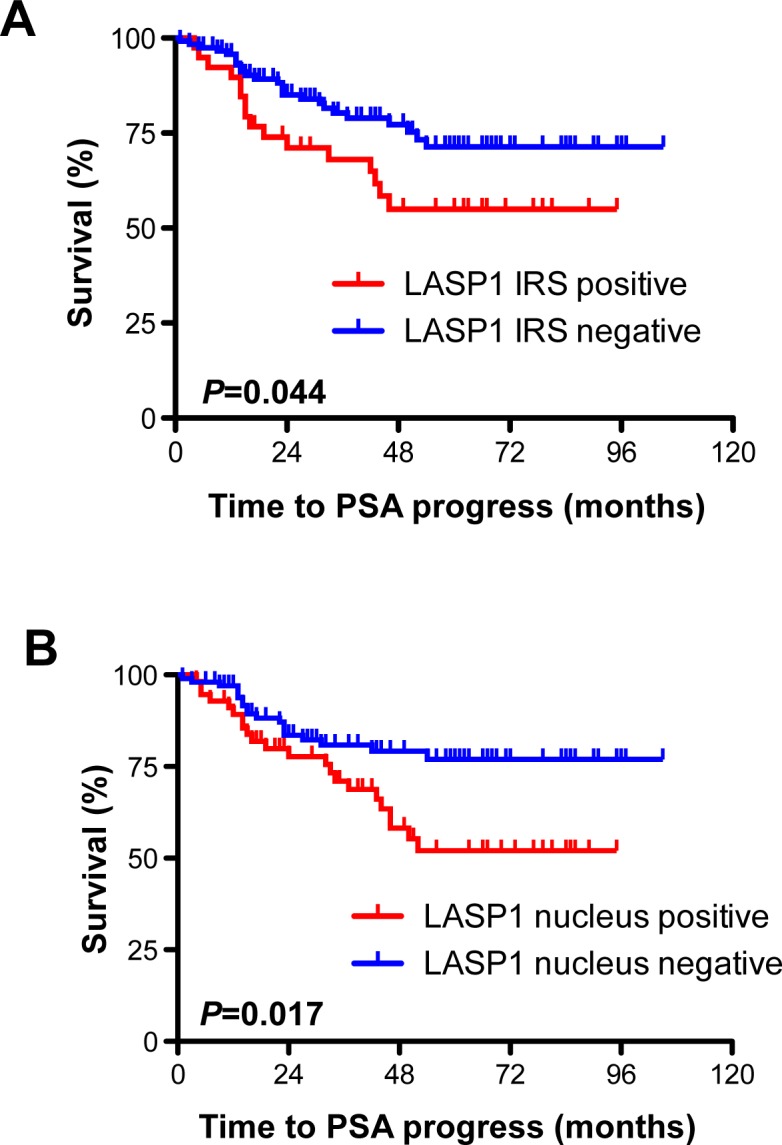
Cytosolic and nuclear LASP1 positivity correlate with PSA progress A: Kaplan-Meier plot displaying patients' probability for PSA progress stratified by cytosolic LASP1 positivity (IRS>5) and negativity (IRS<5) B: Kaplan-Meier plot displaying patients' probability for PSA progress stratified by nuclear LASP1 positivity (NUC≥10%) and negativity (NUC<10%)

The prognostic impact of cytosolic and nuclear LASP1 immunoreactivity was tested using Kaplan-Meier survival analyses. We found a significant correlation between PSA recurrence, both, with nuclear LASP1 positivity (p=0.017) and with strong LASP1 IRS (p=0.044) (Figure 4). These statistical associations were confirmed by univariate analysis (Table 3). For cancer related death (CRD), we failed to attend significance between cytosolic LASP1 levels and this parameter (p=0.1, Table 3). However, the overall number of deceased persons (n=10) was too low for a statistically robust conclusion on CRD (Table 1). Univariate analysis of the data revealed no correlation between Gleason score and high cytosolic or nuclear LASP1 immunoreactivity (Table 3). In synopsis, LASP1 protein expression correlates with PSA progress.

**Table 3:** Univariate analysis of positive nuclear LASP1 localisation and cytosolic LASP1 staining with clinico-pathological parameters (n=161)

Parameters (No. patients)	Positive nuclear LASP1 (≥10%)	p-value	Positive cytosolic LASP1 (IRS >5)	p-value
Nodal status N+ (59) N− (102)	16 (27.1%) 40 (39.2%)	0.13 (F)	7 (11.9%) 32 (31.4%)	0.007 (F)
Tumour size pT2 (23) pT3a (44) pT3b (67) pT4 (27)	11 (47.8%) 16 (36.4%) 19 (28.4%) 10 (37.0%)	0.33 (M)	11 (47.8%) 13 (29.5%) 9 (13.4%) 6 (22.2%)	0.012 (M)
Gleason Score 6 (3) 7 (49) 8 (52)9 (43) 10 (14)	1 (33.3%) 18 (36.7%) 24 (46.2%) 9 (20.9%) 4 (28.6%)	0.16 (M)	0 (0.0%) 11 (22.4%) 15 (28.8%) 10 (23.3%) 3 (21.4%)	0.84 (M)
Recurrence (19) PSA progress (41) Cancer related death (10)	5 (26.3%) 22 (53.7%) 3 (30%)	0.35 (LT) 0.02 (LT) 0.54 (LT)	6 (31.6%) 16 (34.1%) 5 (50%)	0.54 (LT) 0.04 (LT) 0.10 (LT)

(F) Fisher's exact test, (M) Mann-Whitney-U-test; (LT) Log-rank test; statistical significance is assumed at p<0.05.

### mir-203 levels are reduced in prostate cancer

**Figure 5:**
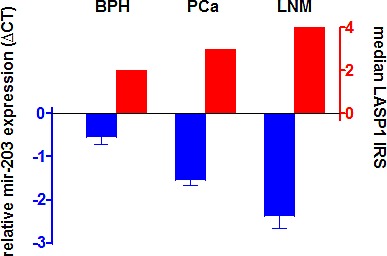
mir-203 levels are reduced in prostate cancer Reduced mir-203 levels correspond to enhanced LASP1 protein concentrations mir-203 expression was analysed by qRT-PCR. Relative mir-203 expression values are presented as mean ± SEM.

To investigate the role of mir-203 on LASP1 expression in PCa, we assessed the mir-203 levels for a subset of our cohort described in Table 2 (15 BPH, 138 high-risk PCa and 12 corresponding PCa/LNM) by qRT-PCR and matched the RNA data with the LASP1 IRS values determined by IHC (Table 2). For PCa a significant correlation between high cytosolic LASP1 protein levels (IRS>5) as well as high nuclear LASP1 levels (NUC≥10%) and reduced mir-203 expression is observed (p=0.002 and p=0.038, respectively). Expression levels of mir-203 are significantly reduced from BPH over PCa (p=0.006) to LNM (p=0.036) while in return LASP1 protein levels are increased, supporting the hypothesis of LASP1 as a potential marker for aggressive PCa (Figure 5).

## DISCUSSION

Current clinical staging is unable to accurately identify PCa subsets that are prone to progress to aggressive lethal disease, even if high Gleason scores and elevated PSA levels are used as combined prognostic markers [25]. This has contributed to a serious dilemma of overtreatment [26]. In the present study we identified LASP1 as a potential new prognostic PCa biomarker since the protein is significantly overexpressed in PCa compared with BPH (p=0.03) in our cohort. Moreover, analysis of 17 specimens of PCa and their corresponding LNM exhibited higher LASP1 levels in metastases, which points to a role of LASP1 in tumor progression. In addition, we analyzed LASP1 expression in high-risk PCa (Gleason score >8 and PSA>20 ng/ml) and correlated the immunoreactive LASP1 scores with clinicopathological data, which yielded a significant correlation between cytosolic and nuclear LASP1 levels and PSA progression. Similarly, a correlation between nuclear LASP1 localisation and poor overall survival is observed in breast cancer [11]. For colorectal cancer [17] and medulloblastoma [15] no distinct differentiation between cytosolic and nuclear LASP1 positivity was performed but patient survival was again inversely correlated with global LASP1 expression. Unexpectedly, no correlation with clinical progression is observed in our study (Table 3). However, on average it lasts 8 years from PSA progression to clinically overt metastasis [27]. Our median study follow up was 4 years, which might explain, at least in part, the lack of statistically association of LASP1 levels with clinical progression in our cohort.

In support of our observations in primary PCa, we found a functional role of LASP1 in LNCaP cells. Besides a moderate reduction of cell proliferation, we observed an impaired migration of LNCaP cells upon inducible shRNA-mediated LASP1 knockdown. These *in vitro* results correspond to the *in silico* predictions derived from our GSEA of microarrays of primary PCa, which showed an association of LASP1 expression with transcriptional signatures involved in locomotory pathways. In analogy, reduced cellular migration upon LASP1 knockdown was observed in medulloblastoma [15], breast cancer [24], and colorectal cancer [17]. Consistently, in several cancer entities such as breast cancer [20], bladder cancer [28], squamous cell carcinoma [19] and now PCa, increased LASP1 protein levels are connected to reduced mir-203 RNA levels and concomitant enhanced cell proliferation and migration.

Notably, in parallel to our experiments, Erho et al. identified LASP1 as one out of 22 marker in a PCa genomic classifier (GC) [8]. The study revealed that 60% of clinical high-risk patients would be reclassified as low-risk with a cumulative incidence of metastasis of only 2.4% at 5 years post radical prostatectomy. Conversely, patients with the highest GC score had nearly 10 times higher cumulative incidence of metastasis by 5 years. The value of this GC in routine clinical practice was assessed in two additional studies [29] [30].

In summary, our results suggest that LASP1 overexpression, most likely mediated by the loss of mir-203 expression, is involved in progression and metastasis of PCa. These data add further functional support for LASP1 being part of the new GC set that discriminates indolent from more aggressive PCa subtypes.

## METHODS

### Tissue samples and study population

Patients' clinicopathologic characteristics are summarized in Table 1. 161 archived paraffin-embedded tissue samples from human prostate cancer (PCa) with confirmed histological diagnoses (radical prostatectomy), 17 corresponding lymph node metastases (LNM) and samples of 15 benign prostatic hyperplasia (BPH) were obtained from the Department of Pathology of the University of Karlsruhe, Germany [31]. All studies were performed with the approval of the Institutional Review board of the Universities of Wuerzburg and Karlsruhe and complied with national laws and the declaration of Helsinki. Grading of PCa malignancy was evaluated according to the Gleason score [32]. Tumor staging was conducted according to parameters of the TNM classification system [4]. Follow-up was performed every 3 months for the first 2 years after surgery, every 6 months in the following 3 years, and annually thereafter. Clinical recurrence is the clinical failure after prostatectomy defined either as histologically proven local recurrence or distant metastasis confirmed by a CT or bone scan that had the date of failure. Biochemical progression/PSA progression was defined as PSA ≥0.2 ng/ml on 2 consecutive follow-up visits.

### Microarray and gene-set enrichment analyses (GSEA)

To compare LASP1 mRNA expression in malignant and normal prostate tissue publicly available microarray data of primary (n=61) and metastatic PCa (n=25) as well as normal prostate tissue (n=18) were retrieved from the Gene Expression Omnibus (GEO; accession numbers: GSE6604, GSE6605, GSE6606, Affymetrix HG-U95Av2 arrays). In addition a much larger publicly available gene expression data of a study analyzing n=154 individual PCa samples was retrieved from the GEO (accession number: GSE17951, Affymetrix HG-U133Aplus2.0 microarrays) for pathway analyses. Expression data were manually revised for their correct annotations and simultaneously normalized by Robust Multi-array Average (RMA) [33, 34] using custom brainarray (v15 and v17 ENTREZG) CDF files yielding one optimized probe-set for each gene corresponding to the ENTREZ gene ID as described elsewhere [35]. To identify pathways and biological processes associated with LASP1 overexpression in PCa we applied a gene-set enrichment analysis (GSEA) on the normalized microarray data [36]. GSEA was performed with 1000 permutations using a pre-ranked list composed of the log2-transformed fold changes of the median gene expression values comparing the top 10 LASP1-high with the top 10 LASP1-low PCa samples (Supplemental Table 1).

### Immunohistochemistry

For immunostaining, sections were placed onto SuperFrost® slides (Langenbrinck, Emmendingen, Germany), dewaxed in xylene, rehydrated in graded ethanol and in dH_2_0. For antigen retrieval, sections were subjected to heat pre-treatment by boiling in 0.01 M of sodium citrate buffer (pH 6.0) for 10 min in a microwave oven (750 Watt/sec.). Endogenous peroxidase was blocked by incubation in 0.1% hydrogen peroxide in PBS for 5 min. Slides were then incubated with the polyclonal anti-LASP1 antibody [37] diluted 1:1000 in “antibody diluent” (DAKO, Hamburg, Germany) followed by EnVision/rabbit detection system (DAKO, Hamburg, Germany). All immunohistological samples were evaluated by two independent scientists for defining of the percentage of LASP1 positive cells and the cytosolic immunoreactivity. Scoring of cytosolic LASP1 expression was carried out in analogy to the scoring of the hormone receptor Immune Reactive Score (IRS), ranging from 0-12 according to Remmele et al. and is described in detail for LASP1 in breast cancer [12, 38]. For better statistical discrimination, samples scored with cytosolic LASP1-IRS <5 were classified as LASP1-negative and those with LASP1-IRS >5 as LASP1-positive. Nuclear LASP1 positivity: Nuclear LASP1 positivity was scored by determining the percentage of positive nuclei regardless of cytosolic LASP1 immunoreactivity. Samples were considered as nuclear-positive if 10% or more cells showed nuclear LASP1 staining.

### Cell lines and culture conditions

PCa cell line LNCaP derived from a lymph node metastasis was purchased from American Type Culture Collection (ATCC, Manassas, USA). Cells were grown in plastic cell culture flasks at 37°C under 5% CO_2_ atmosphere in RPMI 1640 medium (Life Technologies, Darmstadt, Germany) containing 10% heat-inactivated fetal bovine serum (FBS), 1% penicillin/streptomycin, 1% non-essential amino acids and 1% pyruvate (all Invitrogen, Darmstadt, Germany). Mycoplasma contamination was ruled out by PCR.

### mir-203 - qRT-PCR

Total RNA was extracted from PCa, LNM and BPH tissues with Total RNA Extraction Kit (Life Technologies). The RNA concentration was determined with a Bioanalyser (Biorad, Munich, Germany). cDNA was synthesized according to the TaqMan miR Assay protocol (Life Technologies). Mature mir expression was quantified in tissue samples with TaqManR mir assay kits and an Applied Biosystems 7900 HT system. We followed the protocol provided in the manufacturer's instruction (Applied Biosystems, Foster City, CA, USA). The expression of RNU6B was used for normalization. Relative mir expression was calculated with the Δ*C*t-method (Δ*C*t sample = *C*t RNU6B *- C*t sample). Calculations were carried out assuming equal RNA-concentrations and complete efficacy of qRT-PCR.

### RNA interference

For generation of an inducible LASP1 knockdown, LNCaP cells were infected with lentivirus (MOI: 1:10) containing a pTRIPZ vector with either a short hairpin RNA (shRNA) against LASP1 (clone V2THS-64686 mature antisense sequence 5'-GGCAAGTGGAATATCTTATAT-3', Thermo Scientific) or respective non-targeting control shRNA. Successfully transduced LNCaP were selected in 0.5 μg/ml puromycin (Invitrogen). Knockdown efficiency upon doxycycline-treatment (0.5μg/ml) was confirmed by WB.

*Lentivirus production:* 5.5×10^6^ HEK293T cells were seeded into a 100 mm cell culture dish coated with 0.01% poly-L-lysine (Sigma-Aldrich, Deisenhofen, Germany) one day prior to transfection and cultured in DMEM (Invitrogen) supplemented with 10% FBS, 1% penicillin/streptomycin (both Invitrogen). Arrest-In™ (Thermo Scientific) was used as transfection reagent. DNA/Arrest-In™ complexes were formed by mixing 9 μg of the particular pTRIPZ vector DNA, with 28.5 μg of optimized packaging plasmid mix (pTLA1-Pak, pTLA1-Enz, pTLA1-Env, pTLA1-Rev and pTLA1-TOFF, all Open Biosystems, Thermo Scientific) in 1ml DMEM with 187.5 μg Arrest-In™ diluted in 1ml DMEM. Supernatant was harvested 48h and 72h after transfection and lentiviral particles were isolated by filtration and subsequent ultracentrifugation.

### Western blot (WB)

Cells were lysed in Laemmli-buffer containing 10% β-mercaptoethanol (Sigma-Aldrich). Equal amounts of cells were resolved by 10% SDS-PAGE. After blotting on a nitrocellulose membrane (Schleicher&Schuell, Dassel, Germany) the membrane was blocked with 3% nonfat dry milk (Biorad) in TBS-T buffer (10 mM TRIS, 150 mM NaCl, 0.1% (w/v) Tween, pH 7.5). Then the membrane was incubated with a self-generated primary antibody against LASP1 [35] diluted 1:8000 and anti-β-Actin by Santa Cruz (Santa Cruz, CA, USA) diluted 1:2000. Finally the membrane was washed with TBS-T and incubated with the secondary antibody goat-anti-rabbit horseradish peroxidase-coupled and diluted 1:5000 (Biorad). The amount of detected protein was visualized by enhanced chemiluminescence (Amersham Biosciences, Freiburg, Germany) and autoradiography. Quantification of autoradiography signals was carried out by densitometry using the ImageJ software (NIH, Bethesda, USA).

### Proliferation assays

Non-transduced LNCaP cells and cells stable transduced with control shRNA or shRNA against LASP1 were seeded in 48-well plates. Per well 1×10^4^ cells were seeded. After 24 h, medium was replaced by medium +/− doxycycline (0.5 μg/ml). 96h after knockdown induction, cells were counted with CellTiter-Glo ® Luminescent Cell Viability Assay (Promega) following manufacturer's instruction. Assays were performed in 5 independent experiments, each with 6 replicates. In addition, the experiment was performed in T25 flasks and cells were counted with Neubauer chamber. Knockdown of LASP1 was confirmed in each experiment by WB.

### Adhesion assay

To assess cell adhesion non transduced LNCaP cells and cells stable transduced with control shRNA or shRNA against LASP1 were grown for 96h in media (as described previously) +/− doxycycline (0.5μg/ml). Cells were seeded in 48-well plates, per well 4×10^4^ cells in 100μl media. Cells were allowed to attach for 7.5 h at 37°C (ca. 50% adhesion of control cells). In 5 of 8 wells, non-adherent cells were removed by gentle washing with PBS and wells were refilled with 100 μl medium. Wells with non washed-off cells served as 100% value of seeded cells. Cells were counted with CellTiter-Glo ® Luminescent Cell Viability Assay (Promega) following manufacturer's instruction. Assays were performed in 6 independent experiments, (each with 5 replicates). Knockdown of LASP1 was comfirmed in each experiment by WB.

### Migration assay

Cellular migration was assessed by a modified Boyden chamber assay (transwell chambers, Corning Star, Cambridge, MA, USA). Cells were serum-starved overnight, trypsinized, adjusted for viability, counted, and re-suspended in serum-free medium to a concentration of 1×10^6^ cells/ml. Before the experiment, the lower surface of the filter membrane (8 μM pore size) was coated for 15 min with 100 μl fibronectin solution (5 μg/ml; Sigma-Aldrich) as a chemo-attractant. The inner filter chambers were coated with 100 μl 10% FBS in RPMI medium for 30 min. 100 μl cell suspension was placed in the upper filter chambers. The chambers were placed in 24-well plates and cultured in 500 μl RPMI medium with 10% FBS for 4 h at 37°C to allow the cells to migrate through the porous membrane. Non-migrated cells from the top surface were removed using a cotton swab. Migrated cells at the lower surface of the membranes were stained in 200 μl 1% (w/v) crystal violet in 2% ethanol in a 24-well plate for 30 sec and rinsed twice afterwards in distilled water. Cell-associated crystal violet was extracted by incubating the membrane in 200 μl 10% acetic acid for 20 min and measured at 595 nm absorbance using a plate reader (Molecular Devices, Crawley, UK). Four independent experiments, each with 6 replicates, were performed

## SUPPLEMENTARY INFORMATION


